# Neocortical substrates of feelings evoked with music in the ACC, insula, and somatosensory cortex

**DOI:** 10.1038/s41598-021-89405-y

**Published:** 2021-05-12

**Authors:** Stefan Koelsch, Vincent K. M. Cheung, Sebastian Jentschke, John-Dylan Haynes

**Affiliations:** 1 Department of Biological and Medical Psychology, University of Bergen, Bergen, Norway; 2 Department of Neuropsychology, Max Planck Institute for Human Cognitive and Brain Sciences, Leipzig, Germany; 3 Institute of Information Science, Academia Sinica, Taipei, Taiwan; 4 Department of Psychosocial Science, University of Bergen, Bergen, Norway; 5 Berlin Center for Advanced Neuroimaging, Charité - Universitätsmedizin Berlin, Berlin, Germany

**Keywords:** Emotion, Neuroscience, Cortex

## Abstract

Neurobiological models of emotion focus traditionally on limbic/paralimbic regions as neural substrates of emotion generation, and insular cortex (in conjunction with isocortical anterior cingulate cortex, ACC) as the neural substrate of feelings. An emerging view, however, highlights the importance of isocortical regions beyond insula and ACC for the subjective feeling of emotions. We used music to evoke feelings of joy and fear, and multivariate pattern analysis (MVPA) to decode representations of feeling states in functional magnetic resonance (fMRI) data of *n* = 24 participants. Most of the brain regions providing information about feeling representations were neocortical regions. These included, in addition to granular insula and cingulate cortex, primary and secondary somatosensory cortex, premotor cortex, frontal operculum, and auditory cortex. The multivoxel activity patterns corresponding to feeling representations emerged within a few seconds, gained in strength with increasing stimulus duration, and replicated results of a hypothesis-generating decoding analysis from an independent experiment. Our results indicate that several neocortical regions (including insula, cingulate, somatosensory and premotor cortices) are important for the generation and modulation of feeling states. We propose that secondary somatosensory cortex, which covers the parietal operculum and encroaches on the posterior insula, is of particular importance for the encoding of *emotion percepts*, i.e., preverbal representations of subjective feeling.

## Introduction

Which brain areas encode feeling states? Current neurobiological emotion theories posit that, while limbic and paralimbic “core structures”^1,2^, or “survival circuits”^3^, generate emotions (or constitute “core affect”)^4^, neocortical regions represent feelings^5^. In particular the insula, in conjunction with isocortical (visceromotor) anterior and dorsal cingulate regions^6,7^, has been implicated in interoceptive and visceromotor functions: The posterior insula is conceived of as *primary interoceptive cortex*^8^, which provides a representation of the physiological condition of the body, and the anterior insula is implicated in the integration of visceral and somatosensory information with vegetative activity^6,7,9,10^. Recent advances, however, suggest that the encoding of feelings encompasses additional neocortical regions beyond the insula and cingulate cortex. For example, it has been suggested that feelings are states of consciousness, and are therefore expected to be represented in “cognitive workspace circuits”^3^, and it has been proposed that emotional “conceptualization” (“the process by which sensations from the body or external world are made meaningful”)^4^ is represented in neocortical regions such as dorsomedial prefrontal cortex and areas 23 and 31 of the posterior cingulate cortex^4^. Our own group suggested that secondary somatosensory cortex (SII) is a neural correlate of feeling states (or “emotion percepts”), due to its anatomical connections to primary sensory and interoceptive cortex as well as to subcortical structures (thus having access to sensorimotor, exteroceptive, proprioceptive, interoceptive, and limbic information)^5^. However, there is lack of knowledge about (and therefore little agreement on) brain regions encoding feeling states beyond the insula and (anterior) cingulate cortex.

The present study addresses this knowledge gap by using multivariate pattern analysis (MVPA) of functional magnetic resonance imaging (fMRI) data to identify brain regions that encode emotional states. So far, the vast majority of functional neuroimaging studies investigating emotion have used mass-univariate encoding models (of around 8000 published fMRI studies on this topic to date, fewer than 80 studies, i.e. less than 1 percent, have used MVPA). Notably, MVPA complements univariate approaches because it can make use of more information contained in spatially distributed fMRI signal patterns than univariate approaches^11^. Notably, while mass-univariate encoding models find predominantly limbic and para-limbic regions in emotion experiments^12,13^, studies investigating emotions using MVPA find activity changes mainly in neocortical regions (see also below for details). For example, it was found that feelings of six basic emotions could be predicted based on fMRI signals in somatosensory, premotor, fronto-median, and lateral frontal cortex, while signals from the amygdala provided only little predictive information^14^. That finding corroborated the notion that limbic/paralimbic structures organize basic functions related to arousal, saliency, and relevance processing (which are engaged for all emotions), while discrete emotional states, and in particular subjective feelings, are the result of interactions between limbic/paralimbic structures on the one hand, and neocortical structures on the other^1,3–5,14^.

In the present study we used music to evoke feelings of joy and fear. We chose music due to its power to evoke strong emotions (and, therefore, strong subjective feelings as one of the sub-components of emotion)^15^. However, only very few decoding studies have used music to investigate emotion. One study used a whole-brain searchlight analysis to decode processing of brief bursts of auditory emotions (about 1.5 s long, produced by instruments or vocalizations)^16^. That study reported that BOLD signals in primary and secondary auditory cortex (AC), posterior insula, and secondary somatosensory cortex (parietal operculum) predicted the affective content of sounds, both within and across domains (instruments, vocalizations). Another decoding study with music manipulated the temporal structure of music and speech, comparing original music with scrambled stimuli (scrambled music being both less intelligible and less pleasant than unscrambled music)^17^. That study reported regions with significant decoding accuracy in the auditory cortex, insular cortex, and frontal operculum. Moreover, in a cross-modal decoding study using music and videos, voxels in the superior temporal sulcus (STS) were found to be sensitive to the valence of both music and videos^18^. The regions found in these studies were also observed in studies using decoding approaches to localize regions representing vocal-affective processing^19–21^: regions with above-chance emotion decoding accuracy were found in the auditory cortex, including the superior temporal gyrus (STG) and the STS, as well as the right anterior insula and right fronto-opercular areas. Note that, beyond the auditory cortex, those decoding results were unlikely to be driven merely by acoustical features of stimuli, because studies that have used music to study the decoding of acoustical features reported that the classifiers mainly included voxels in the auditory cortex (these studies include the decoding of acoustical descriptors, musical genres, individual speakers and instruments, pitch intervals, absolute pitch, or musical experience)^22–29^.

Interestingly, one of these studies also investigated the time course of decoding accuracy^24^, finding that already after a few seconds predictions for different musical genres were ~ 65% accurate, increasing only moderately (i.e., up to ~ 70%) after 17 s. The finding of early (within seconds) classification accuracy for musical information corresponds to the observations that emotions expressed in music are recognized, and elicit emotion-specific peripheral-physiological responses, within a few seconds^30,31^. Neuroimaging data suggest that the auditory cortex, amygdala, and nucleus accumbens show quick responses to musical stimuli (within about 10 s after stimulus onset)^32–34^, while the somatosensory cortex comes into play at later stages (within about 20 s)^34^. However, only few studies have investigated the time-course of brain activity in response to musical stimuli, and, therefore, knowledge about this issue is still tentative. This knowledge-gap led us to analyze the time course of decoding accuracy in our study.

To generate specific hypotheses for the present study, we performed a preparatory decoding analysis of fMRI data from a previously published fMRI experiment^34^. That study used music to evoke feelings of joy or fear, but only reported a univariate (general linear model) contrast analysis. Before carrying out the current experiment, we performed a decoding analysis of those published data. That decoding analysis indicated two large clusters with local maxima bilaterally in the right and left auditory cortex (including STG and STS), the posterior insula, and the parietal operculum (Supplementary Fig. S1). In addition, smaller clusters were indicated in the left frontal operculum, the right central sulcus (premotor and primary somatosensory cortex, with a local maximum in the region of the face area), and the right posterior middle temporal gyrus (MTGp).

Motivated by these results, we designed a new experiment with a similar experimental design, but specifically tailored for a decoding analysis (see “Methods”). Based on the preparatory decoding analysis and the decoding studies on emotions using music or voices (as reported above), we hypothesized that informative regions for the feeling states of joy and fear would be located in the STG and STS (auditory cortex), posterior insula, parietal operculum (secondary somatosensory cortex), pre- and postcentral gyri (premotor cortex and primary somatosensory cortex in the region of the face area), the frontal operculum, and MTGp. In addition, to investigate how feeling representations develop over time, we segmented our 30 s stimuli into five equal segments (each 6 s long). No specific hypotheses were made regarding the time course of decoding accuracy, due to the scarcity of studies on this issue (as reviewed above).

## Methods

### Participants

Twenty-four individuals (13 females; age range 20–34 years, *M* = 22.79, *SD* = 3.45) took part in the experiment. All participants gave written informed consent. The study was conducted in accordance with the declaration of Helsinki and approved by the Regional Committee for Medical and Health Research Ethics West-Norway (reference nr. 2018/363). Exclusion criteria were left-handedness, professional musicianship, specific musical anhedonia (as assessed with the Barcelona Music Reward Questionnaire)^35^, past diagnosis of a neurological or psychiatric disorder, significant mood disturbances (a score of ≥ 13 on the Beck Depression Inventory)^36^, excessive consumption of alcohol or caffeine during the 24 h prior to testing, and poor sleep during the previous night. All participants had normal hearing (assessed with standard pure tone audiometry). None of the participants was a professional musician, and participants received on average *M* = 4.41 years of extracurricular music lessons (0–1 year: *N* = 10 participants, 1–5 years: *N* = 6, more than 5 years: *N* = 8); half of the participants played an instrument or sang regularly at the time they participated.

### Stimuli

Twelve musical stimuli, belonging to two categories, were presented to the participants: Six stimuli evoked feelings of joy, the other six feelings of fear (Supplementary Table S1). The stimuli were identical with those used in a previous study^34^, except that no neutral stimuli were included and the number of stimuli in each category was reduced from eight to six (this was done to optimize the experimental paradigm for a decoding approach). Joy excerpts were taken from CD-recorded pieces from various styles (soul, jazz, Irish jigs, classical, South American, and Balkan music). Fear stimuli were excerpts from soundtracks of suspense movies and video games. Joy and fear stimuli were grouped into pairs, with each pair being matched with regard to tempo, mean fundamental frequency, variation of fundamental frequency, pitch centroid value, spectral complexity, and spectral flux. The acoustic dissonance of the fear stimuli, on the other hand, was electronically increased to increase the fear-evoking effect using Audacity (https://www.audacityteam.org; for details cf. Ref.^34^). The control of acoustical and musical features differing between the music conditions is described in detail further below. Also using Audacity, all stimuli were compressed, normalized to the same RMS power, and cut to the same length (30 s with 1.5 s fade-in/fade-out ramps). Compression served to avoid perception difficulties due to the scanner noise by reducing the dynamic range of the audio-stimuli (i.e., the difference between the loudest and the softest part) with a threshold of 12 dB, noise floor -40 dB, ratio 2:1, attack time 0.2 s and release time 1.0 s.

### Acoustical feature analysis

Although each joyful and fearful stimulus-pair was chosen to match in several acoustical and musical features (as described above), we extracted 110 acoustic features, using the *Essentia* music information retrieval library (https://www.essentia.upf.edu) in order to control for acoustical features that differed between joy and fear stimuli. The extracted features included spectral, time, rhythmic, pitch, and tonal features that are used to describe, classify, and identify audio samples. Each stimulus was sampled with 44,100 Hz, and frame-based features were extracted with a frame and hop size of 2048 and 1024 samples, respectively. We chose those acoustical features that differed significantly under a threshold of *p* < 0.05 after correcting for multiple comparisons with Holm’s method. Three sensory-acoustic features were indicated to differ between joy and fear stimuli: mean spectral complexity, mean sensory dissonance, and variance in sensory dissonance (spectral complexity is related to the number of peaks in the spectrum of an auditory signal, and sensory dissonance is a measure of acoustic roughness in the auditory stimuli). These three acoustic features where then entered in the fMRI data analysis as regressors of no interest (see below for details).

### Procedure

Before scanning, participants filled out the questionnaires, underwent a standard audiometry, and were trained in the experimental procedure. During the fMRI experiment, participants were presented with 5 blocks of stimuli. Within each block, all twelve stimuli were presented in pseudo-randomized order so that no more than two stimuli of each category (joy and fear) followed each other. Participants were asked to listen to the music excerpts with their eyes closed. At the end of each musical stimulus, a beep tone (350 Hz, 1 s) signaled participants to open their eyes and perform a rating procedure. Each rating procedure included four judgements, to assess four separate dimensions of their feelings: Participants indicated how they felt at the end of each excerpt with regard to valence (“How pleasant have you felt?”), arousal (“How excited have you felt?”), joy (“How joyful have you felt?”), and fear (“How fearful have you felt?”). Participants were explicitly instructed to provide judgements about how they felt, and not about which emotion they recognized to be expressed by a stimulus. Judgements were obtained with six-point Likert scales ranging from “not at all” to “very much”. For each rating procedure, the order of presentation of the four emotional judgements was randomized to prevent motor preparation. For the same reason, and to balance motor activity related to the button presses, the polarity for each rating scale was randomized (with each rating polarity having a probability of 50%). For example, in one rating procedure they had to press the outermost *left* button, and in another rating procedure the outermost *right* button, to indicate that they felt “very pleasant”. Participants were notified of the rating polarity at the beginning of each rating procedure (this information indicated whether the lowest value of the scale was located on the left or on the right side). Thus, the polarity of each scale was only revealed *after* each musical stimulus (unpredictably for the subject). Moreover, the order of ratings changed unpredictably (for example, in one trial starting with the valence judgement, and in another trial with the arousal judgement etc.). Therefore, subjects were not able to prepare any motor responses for the rating procedure during listening to the musical stimulus. Participants performed the ratings using response buttons in their left and right hand.

Each trial lasted 53 s. It began with the musical excerpt (30 s), followed by the rating procedure (23 s). Within the rating procedure, the instruction screen showing the rating polarity was shown for 3 s, followed by 4 ratings (each 4 s), and concluded with a pause of 4 s. Each of the five blocks contained 12 trials (10′36′’ in total). Between blocks was a pause of about 1 min during which the scanner was stopped to avoid any temporal correlations between blocks.

The experiment was carried out using E-prime (version 2.0; Psychology Software Tools, Sharpsburg, PA; www.pstnet.com). Auditory stimuli were presented using MRI compatible headphones (model NNL HP-1.3) by Nordic NeuroLab (NNL, Bergen, Norway), with a flat range response of 8 to 35,000 Hz, and an external noise attenuation of an A-weighted equivalent continuous sound level of 30 dB. Instructions and rating screens were delivered using MRI compatible display goggles (VisualSystem by NNL). Synchronization of stimulus presentation with the image acquisition was executed through an NNL SyncBox.

### Data acquisition

MR acquisition was performed with a 3 T GE Signa scanner with a 32-channel head coil. First, a high-resolution (1 × 1 × 1 mm) T1-weighted anatomical reference image was acquired from each participant using an ultra-fast gradient echo (FSPGR) sequence. The functional MR measurements employed continuous Echo Planar Imaging (EPI) with a TE of 30 ms and a TR of 2100 ms. Slice-acquisition was interleaved within the TR interval. The matrix acquired was 64 × 64 voxels with a field of view of 192 mm, resulting in an in-plane resolution of 3 mm. Slice thickness was 3 mm (37 slices, with 0.6 mm interslice gap, whole brain coverage). The acquisition window was tilted at an angle of 30° relative to the AC-PC line in order to minimize susceptibility artifacts in the orbitofrontal cortex^37^. To gain enough data points for analysis and given stimulus duration (30 s), a continuous scanning design was employed.

### Data analysis

Behavioral data and participant characteristics were analyzed using SPSS Statistics 25 (IBM Corp., Armonk, NY). The emotion ratings were evaluated with MANOVAs for repeated measurements with the factors condition (“fearful” vs. “joyful”), block (5 levels for 5 blocks), and pair (6 levels for the six stimulus pairs). Where necessary, results were corrected for multiple comparisons using Bonferroni-correction.

### MRI analysis: main decoding analysis

Acquired functional data were analyzed using SPM 12 (Welcome Trust Centre for Neuroimaging, London, UK) and MATLAB 2017b (The MathWorks, Inc., Natick, MA, USA). The images were first despiked using 3dDespike in AFNI^38^, slice timing corrected, and corrected for motion and magnetic field inhomogeneities for preprocessing. No spatial normalization or smoothing were applied at this stage to preserve fine-grained information in local brain activity^39^. A single general linear model was then estimated for each participant. For each of the five runs, joy and fear stimuli were modelled as two boxcar regressors (duration = 30 s) with parametric modulators adjusting for significant differences in valence ratings and standardized sensory-acoustic features (mean spectral complexity, mean sensory dissonance, and variance in sensory dissonance, as described above) across stimuli from the two emotion categories. An event-related impulse was also included to model each finger-press. These regressors were convolved with the canonical haemodynamic response function. Six rigid-body transformation regressors were then added to reduce motion-induced artefacts. Temporal autocorrelation in the time-series were captured using a first-order autoregressive model, and low-frequency scanner drifts were removed by high-pass filtering with a 128 s cutoff.

Multivariate pattern analysis was carried out on the subject level using The Decoding Toolbox (TDT) 3.99^40^. For each voxel in the brain, a spherical searchlight with a radius of 3 voxels was defined. A linear support vector machine with regularization parameter C = 1 was trained and tested on run-wise parameter estimates of joyful and fearful stimuli for voxels within each searchlight using leave-one-out cross-validation. Run-wise parameter estimates were used for enhanced stability, the searchlight method was chosen to reduce the dimensionality of classification, and cross-validation was implemented to control for overfitting^11^. Classification accuracy maps from each cross-validation fold were mean averaged for each participant. The resulting maps were then normalized to MNI space and resampled to the native resolution of 3 mm-isotropic^41^. No spatial smoothing was performed to preserve the granularity of our results.

Group-level results were obtained through permutation-based t-tests and corrected for multiple comparisons using LISA^42^. LISA is a threshold-free correction method that utilizes a non-linear edge-filter to preserve spatial information in fMRI statistical maps and does not require prior smoothing of statistical maps. Compared with other correction methods, LISA has the advantage of preserving spatial specificity and reducing Type II error whilst simultaneously maintaining Type I error control. Note that standard *t*-tests on decoding accuracies do not provide inference on the population level^43^. A voxel-wise false discovery rate-corrected threshold of *p* = 0.05 was adopted, and anatomical regions were identified using the SPM Anatomy Toolbox 2.2c^44^. Note that, because statistical significance was computed on the voxel level, and not on the cluster level, each significant voxel provides sufficient decoding information.

### MRI analysis: temporally segmented decoding analysis

Given the relatively long duration of our stimuli, we were interested to see how the encoding of information between joyful and fearful music differed at various time points during stimulation. The analysis pipeline was similar to the main decoding analysis. However, joyful and fearful stimuli were now divided into five equal segments of 6 s, with each segment modelled separately using boxcar regressors and parametric modulators when estimating the general linear model for each subject. Multivariate decoding analyses were subsequently carried out on the parameter estimates of joyful and fearful stimuli for each segment separately. Statistical maps for each segment were computed with a corrected threshold of *p* < 0.05 using LISA as before.

## Results

### Behavioral data

**Figure 1:**
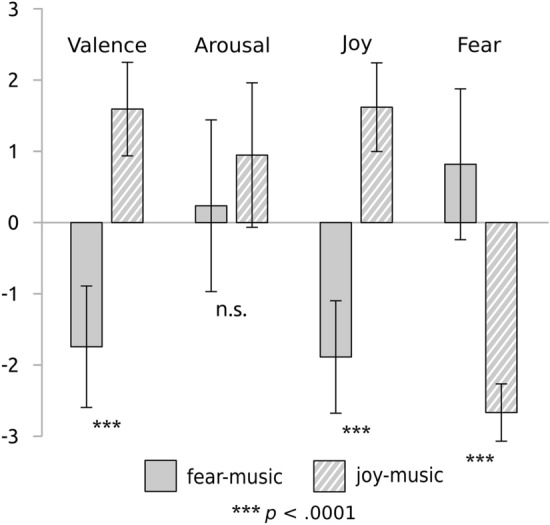
Behavioral ratings. Boxes with error bars (means and standard deviations) indicate the behavioral ratings on the four felt emotion scales: valence (“How pleasant have you felt?”), arousal (“How excited have you felt?”), joy (“How joyful have you felt?”), and fear (“How fearful have you felt?”). Scales ranged from -3 (“not at all”) to 3 (“very much”). Results are shown separately for each stimulus category: ratings for fear-music are indicated by plain grey boxes, and ratings for joy-music by hatched boxes. Joy stimuli evoked markedly stronger feelings of pleasure and joy (compared with fear stimuli), while fear stimuli evoked a stronger feeling of fear (compared with joy stimuli). Arousal did not differ significantly between joy- and fear-stimuli.

Participants rated their feeling states on four scales (valence, arousal, joy, and fear). These ratings are summarized in Fig. 1 and Supplementary Table S2. Joy-music was rated as more pleasant, evoking more joy and less fear than fear-music (*p* < 0.0001 in each *t*-test). The difference in felt arousal was statistically not significant (*p* = 0.07).

### fMRI decoding results

**Figure 2:**
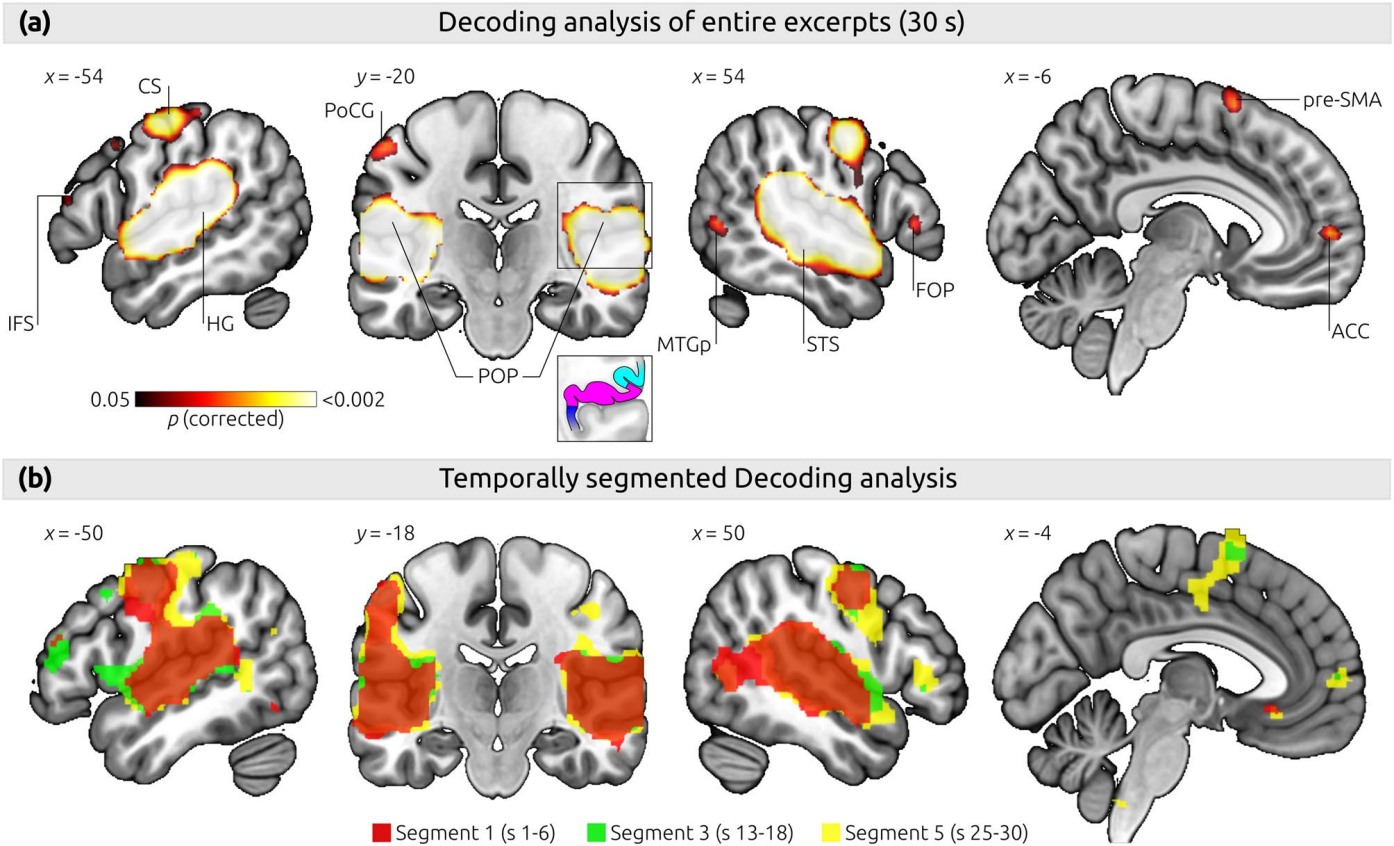
Results of the decoding analysis, showing clusters with voxels providing significant information about the difference between joy- and fear-music (after permutation-based *t*-tests and correction for multiple comparisons, all results were obtained without spatial smoothing, and statistical significance was computed on the voxel level). Results shown in the upper panel **(a)** were obtained using the entire duration of each music stimulus (each stimulus had a duration of 30 s). Significant clusters were found in the auditory cortex (including the superior temporal gyrus and superior temporal sulcus), posterior insular cortex, secondary somatosensory cortex (parietal operculum), primary somatosensory cortex and premotor cortex (post- and precentral gyrus as well as pre-SMA), frontal operculum and inferior frontal sulcus, as well as the ACC. The square in the coronal view indicates the area of the inset underneath; the inset illustrates anatomical boundaries between insula cortex (blue), cortex of the parietal operculum (magenta), and inferior parietal cortex (cyan), according to Ref.^50^. Note that the acoustical features that differed between joy and fear stimuli were entered as regressors of no interest in the data analysis, to reduce the influence of acoustical differences between the stimuli on the results. The lower panel **(b)** shows the temporally segmented results, for the beginning of excerpts (first 6 s, red), middle of the excerpts (seconds 13 to 18, green), and the end of excerpts (seconds 25–30, yellow). Note that, in each cluster, the cluster-size increased from the beginning to the end of excerpts, corroborating the findings of the main decoding analysis. *ACC* anterior cingulate cortex; *CS* central sulcus; *FOP* frontal operculum; *HG* Heschl’s gyrus; *IFS* inferior frontal sulcus; *MTGp* posterior middle temporal gyrus; *PoCG* postcentral gyrus; *POP* parietal operculum; *pre-SMA* pre-supplementary motor area; *STS* superior temporal sulcus.

We found voxels with significant above-chance information about the difference between joy- and fear-stimuli in several brain regions (upper panel of Fig. 2 and Table 1, note that statistical significance was computed on the voxel level, not the cluster level, thus each significant voxel provides information to decode between joy-/fear-music; mean decoding accuracy maps are provided in Supplementary Fig. S2): Bilaterally, significant voxels were indicated in the STS, STG (auditory cortical fields TE1-4 according to Refs.^45,46^), the entire posterior insula, the parietal operculum (secondary somatosensory cortex, OP1-4 according to Ref.^47^), and the central operculum (including premotor cortex). In the right hemisphere, significant voxels were also indicated in the dorsal precentral gyrus (caudal PMd according to Ref.^48^, i.e. dorsal PMC, area 6). In the left hemisphere, voxels with significant decoding information were indicated in the dorsal PMC, the dorsal central sulcus, the crown of the postcentral gyrus (area 1) and the postcentral sulcus (area 2). In addition to dorsal premotor regions, several other regions were indicated in the frontal lobe: left inferior frontal sulcus (IFS), left middle frontal gyrus (MFG), left and right superior frontal gyrus, and right inferior frontal gyrus (pars triangularis of the frontal operculum). Beyond the primary and secondary somatosensory regions of the parietal lobe, significant voxels were located in the superior parietal lobule (area 5), the ACC (according to Ref.^49^), and the pre-SMA. Two regions with significant voxels were located in visual areas (in the right posterior middle temporal gyrus/area MT, and in the left fusiform gyrus), and two regions were observed in the cerebellum bilaterally.

**Table 1:** List of clusters of voxels (and local maxima within clusters) carrying significant information about the difference between joy- and fear-music.

Anatomical structure	Cluster nr	MNI coordinate
R STS fundus (TE 4)	1 (2625 vox)	48 − 28 2
R STG (TE 3)		68 − 25 11
R STG (TE 1)		52 − 12 4
R POP (OP 1, SII)		54 − 24 20
R POP (OP 2, SII)		38 − 23 19
R insula (Ig 1)		34 − 24 10
R precentral g. (caudal PMd, area 6)		54 − 4 44
L STS fundus (TE 4)	2 (2206 vox)	− 48 − 22 − 2
L STG (TE 3)		− 62 − 13 − 1
L STG (TE 1)		− 48 − 18 5
L Insula		− 38 − 18 5
L POP (OP 1, SII)		− 54 − 18 14
L POP (OP 2, SII)		− 34 − 26 17
L MFG	3 (235 vox)	− 48 38 20
L IFS		− 46 38 14
L precentral g. (caudal PMd, area 6)	4 (196 vox)	− 48 0 54
L postcentral g. (area 1, SI)		− 51 − 28 59
R SFG	5 (120 vox)	21 32 52
Pregenual ACC/superior medial gyrus (area 32/10)	6 (77 vox)	0 56 5
R cerebellum (lobule IX)	7 (77 vox)	15 − 43 − 43
L cerebellum (lobule VIIb/VIIIa)	8 (43 vox)	− 33 − 58 − 49
R MTGp (area MT, V5)	9 (43 vox)	54 − 64 5
L SFG	10 (42 vox)	− 15 32 56
pre-SMA	11 (38 vox)	− 6 11 65
L middle occipital (fusiform) gyrus	12 (37 vox)	− 48 − 64 − 14
R IFG (pars triangularis, area 45)	13 (32 vox)	51 29 2
L precentral sulcus (PMC, area 6)	14 (27 vox)	− 27 − 1 50
L SPL (area 5)	15 (18 vox)	− 15 − 49 71

*ACC* anterior cingulate cortex; *CS* central sulcus; *FOP* frontal operculum; *HG* Heschl’s gyrus; *IFG* inferior frontal gyrus; *IFS* inferior frontal sulcus; *Ig* granular subregion of the insular cortex; *L* left; *MFG* middle frontal gyrus; *MTGp* posterior middle temporal gyrus; *OP* subregion of the parietal operculum; *PMC* premotor cortex; *PMd* dorsal premotor cortex; *PoCG* postcentral gyrus; *POP* parietal operculum; *pre-SMA* pre-supplementary motor area; *R* right; *SFG* superior frontal gyrus; *SII* secondary somatosensory cortex; *SPL* superior parietal lobule; *STG* superior temporal gyrus; *STS* superior temporal sulcus; *TE* temporal (auditory) subregion.

The results of the temporally segmented decoding analysis are shown in the lower panel of Fig. 2 (showing decoding results for the first, third and fifth segment; the decoding results for all five segments are provided in Supplementary Fig. S3). Significant decoding accuracy in the first segment (i.e., seconds 1–6) is shown in red, in the third segment (seconds 13–18) in green, and in the fifth segment (seconds 25–30) in yellow. Remarkably, in all clusters found in the main analysis (Table 1), the size of the clusters increased across segments. In all clusters, informative voxels were already detected in the very first segment (seconds 1 to 6), although in several areas (pre-SMA, ACC, and left post-central gyrus) the significance was below the applied threshold during the first segment. Also, as in the main analysis, no significant decoding information was found in the amygdala, the hippocampal formation, or the nucleus accumbens, in any of the segments.

## Discussion

Significant voxels providing information about the feeling representations of joy and fear were found in the auditory cortex (including the superior temporal gyrus and the superior temporal sulcus), interoceptive cortex (posterior insula), secondary somatosensory cortex (parietal operculum, POP), primary somatosensory cortex (posterior central gyrus), premotor cortex (dorso-lateral precentral gyrus and pre-SMA), right frontal operculum, and right area MT in the posterior middle temporal gyrus. These results replicate the results obtained from our hypotheses-generating dataset (obtained with an independent sample of subjects; see “Introduction” & Supplementary Fig. S1), and thus show that our results are reliable. Additional areas found in the present analysis, but not in the hypothesis-generating analysis, included the medial frontal gyrus, inferior frontal sulcus, anterior cingulate cortex (ACC), area 5 in the precuneus, and pre-supplementary motor area (pre-SMA).

Before discussing these results, we would like to comment on the finding that some of the clusters identified in our analysis comprised of different brain regions, which could (mistakenly) be interpreted as brain-activation of one brain region spreading spuriously into adjacent regions (e.g., from the auditory cortex into somatosensory cortex). Here, it is important to note that (i) all analyses were performed without spatial smoothing of the fMRI data, and computed in each subject’s native space before normalizing to MNI-space, thus significant voxels were not “smeared” from one large region into another adjacent region by the smoothing procedure; (ii) significant voxels further away from one region than the searchlight radius (15 mm) could not have been influenced by brain activity in that region, and because most brain regions with significant above-chance decoding information were located further away from the auditory cortex than the searchlight radius, including several voxels of the somatosensory cortex, decoding results in those regions could not have been due to brain activity in the auditory cortex; (iii) statistical significance was computed on the voxel level (not on the cluster level, as is often done in univariate fMRI analyses), thus each significant voxel provides information to decode between joy and fear stimuli; (iv) all participants showed decoding accuracy of at least 70% in SII (20 bilaterally and 4 in the left POP only, see Supplementary Fig. S4), showing that the finding of significant voxels in the POP was not simply an artifact of spatial distortions that might have been introduced by the registration and normalization procedures. Therefore, even though the significant voxels in several regions (e.g. STG, insula, and POP) blend into one large cluster, there was significant, sufficient decoding information in each of these areas.

Out of the observed areas (listed in Table 1), the insula, ACC, and secondary somatosensory cortex are of particular interest for subjective feeling. The finding of decoding information in these areas replicates results of our preparatory data analysis (see “Introduction” and Supplementary Fig. S1), and of previous decoding studies on emotion^14,16,17,19–21^. It is well established that feeling states involve interoceptive cortex (in the posterior insula) and the ACC^6–10^, which were both also found in our study. In addition, our findings support the notion that secondary somatosensory cortex (SII) also plays a role in feelings^5^. This role of SII in feelings has received support by a recent meta-analysis on emotions evoked by music, which indicated a peak maximum in the left SII^51^. Further meta-analytic support is provided by an automated analysis for the term “feeling” using the Neurosynth platform (neurosynth.org): This analysis indicates clusters in both the left and right POP, which is the anatomical correlate of SII (at MNI coordinates [− 50, − 24, 26], and [40, − 33, 28], respectively, based on 101 studies). In addition, activations of primary and secondary somatosensory cortex, as well as premotor cortex, have been observed relatively often in emotion studies, owing to the fact that different emotions elicit discernible somatic sensations and motor preparations (reviewed in Ref.^14^). However, and quite surprisingly, the prominent meta-analyses on emotion do not mention somatosensory cortex at all (neither SI nor SII)^1,4,12,13,52^. This discrepancy is likely due to the pitfall that fMRI activations in the POP are often mistakenly reported as activations of the posterior insular cortex: cytoarchitectonically, the inferior boundary of SII is the retro-insular cortex, and the inferior boundary of SII (which is located mainly in the parietal operculum ) transgresses the circular sulcus into the posterior insula (see inset in Fig. 2a)^50^. Thus, the macroanatomical boundary between insula and POP (the circular sulcus) is not the cytoarchitectonic boundary between insular cortex and SII, and the posterior insula is not equivalent to the posterior insular cortex (because part of SII is located in the posterior insula). Therefore, fMRI activations in the posterior insula and adjacent operculum can easily be mislabeled as insular cortex (instead of SII).

SII is sensitive to touch, pressure, vibration, temperature, and vestibular information. Especially its inferior subregions (OP2 and OP3 according to Ref.^50^) are sensitive to “limbic touch” (i.e., soft touching or slow stroking), and the dorsal subregion OP1 is sensitive to pain. Interestingly, OP2 probably exists only in humans (no corresponding field has been found so far in non-human primates)^47^. Moreover, SII is not only activated by touch, but also by observing other individuals being touched, therefore representing a neural correlate contributing to social-empathic processes^53^. Note that SII is located directly adjacent to extero- and proprioceptive cortex (SI) as well as “primary interoceptive cortex” in the posterior insula^8^, having dense (ipsilateral) anatomical connections to areas 1, 2, and 3 of SI, as well as to agranular, dysgranular, and granular insular cortex^54^. In addition, the POP has connections to further limbic/paralimbic regions including the orbitofrontal cortex, several thalamic nuclei, striatum (including the NAc in the ventral striatum), pallidum, hippocampus, and amygdala^54^. Thus, interoceptive information from insular cortex,to my knowledge, somatosensory information from SI, and affective information (“core affect”) from limbic structures converge in SII, and it has previously been suggested that these different sources of affective information are synthesized in SII into an *emotion percept*^5^, i.e., a preverbal representation of subjective feeling.

In humans, subjective feelings are under strong influence of cognitive processes such as deliberate appraisal^5,55^, emotion regulation^56^, or conceptualization^4^, involving a range of cognitive functions such as attention, working memory, long-term memory, action, and language^3–5^. Thus, the numerous isocortical structures underlying these functions also play a role in human emotion. For example, a meta-analysis of human neuroimaging studies on cognitive reappraisal of emotion found significant clusters in the DLPFC, frontal operculum, PMC, and pre-SMA^55^, all of which have anatomical connections with the POP^54^, and were also found to provide significant decoding information in the present study.

With regard to the auditory cortex, we cannot rule out that the contributions of voxels in the auditory cortex were driven, at least in part, by acoustical differences between stimuli. However, recall that we matched our two classes of stimuli on several acoustical characteristics, and that the acoustical features that differed between joy and fear stimuli were entered as covariates of no interest. It is therefore likely that the decoding results in the auditory cortex were, at least in part, due to its role in the generation of feeling representations. The auditory cortex has direct connections with limbic/paralimbic structures such as the amygdala^57,58^, the orbitofrontal cortex^59^, and cingulate cortex^60^, as well as with the POP^54^. Functional connectivity between auditory cortex and the ventral striatum predicts the reward value of music^61^, and such functional connectivity is reduced in individuals with specific musical anhedonia^62^. Moreover, fMRI research has suggested that auditory core, belt, and parabelt regions have influential positions within emotion networks, driving emotion-specific functional connectivity (e.g. larger during joy- than fear-evoking music) with a number of limbic/para-limbic regions such as NAc, insula and ACC^63^. Thus, the auditory cortex is in a central position to generate feeling representations in response to acoustic information. The role of the auditory cortex in generating feeling representations is probably of particular importance when emotional expression with music employs, and exaggerates, acoustical signals of vocal emotional expressions (as is often the case during more naturalistic music listening)^15^.

With regard to the time-course of the decoding accuracy, the temporally segmented analysis showed that, in all clusters, informative voxels were already detected in the very first segment (seconds 1 to 6). Then, with increasing duration of stimulus presentation the size of all clusters, i.e., the size of regions which are informative about emotion representations, increased further (in our study up until the end of stimuli, i.e. up until 30 s). Thus, our results reveal that cortical feeling representations emerge very early, i.e. measurable within the very first seconds after stimulus exposure. This finding is consistent with the swift recognition of emotions expressed in music^30^, and fast emotion-specific peripheral-physiological responses to music^31^.

A surprising finding of our study was the presence of informative voxels in right area MT (located in the posterior middle temporal gyrus), in both the preparatory analysis (Supplementary Fig. S1) and the main results (Fig. 2). In addition, the main results also indicated a cluster in the fusiform gyrus (FG). Area MT is a higher-level visual area involved in motion perception^64^, and the FG hosts areas specialized for the recognition of faces and bodies, including (in concert with the STS) the recognition of people in motion^65^. Our observation of voxels in these visual regions providing information about emotion representations is consistent with previous observations that music listening elicits mental imagery^66,67^, and with the idea that visual imagery is a basic principle underlying the evocation of emotions with music^15^. In our previous fMRI study (also using joy- and fear-evoking music) participants reported different types of visual imagery during fear music (involving, e.g., “monsters”) than during joy music (involving, e.g., “people dancing”)^34^. Thus, it is tempting to speculate that the decoding of emotional states in visual areas was due to visual imagery specific to joy- and fear-evoking music.

The present results reveal, with the exception of two cerebellar clusters, only neocortical structures. Although this is consistent with previous decoding studies on emotion (where feeling states were predominantly decoded from signals originating from neocortical structures)^14,16–21^, this means that feeling states were not decoded from subcortical voxels in our study (in contrast to numerous fMRI studies on music and emotions using mass-univariate approaches)^51^. We presume two main reasons why we did not find significant decoding information in subcortical structures: (1) searchlight decoding typically uses a small sphere (we used a sphere with a 3-voxel radius), thus the sphere crossed the boundary of different subcortical structures (many of which are relatively small in volume), and therefore the classifier was trained on information from multiple neighboring brain regions. This might have led to the classifier not providing significant decoding accuracies. (2) It is also possible that, in the present study, any subcortical activity changes were not strong enough to be detected by a classifier: When performing a univariate analysis of our data, using identical preprocessing and the same statistical parameters, we did not find significant signal changes in subcortical structures (see Supplementary Fig. S5). It is worth noting that this univariate analysis also showed fewer significant voxels in the cortex than the (multivariate) decoding analysis. For example, in the univariate analysis, significant activations were indicated in the right auditory cortex, but not in the adjacent POP, nor in the right insula. This suggests that neocortical encoding of feeling representations can better be detected with decoding approaches.

### Limitations

In the present study we did not find subcortical activations, neither in the multivariate, nor in the univariate analysis. It is a known challenge that the scanner noise impedes the evocation of music-evoked emotions (e.g., because music does not sound as beautiful and rewarding as outside the scanner). Future studies might consider using sparse temporal sampling designs to mitigate this issue, especially in light of our results which suggest that fewer scans per trial can already lead to reliable decoding results (see Supplementary Fig. S3). Another limitation is that some higher-order structural features of our music stimuli might have differed systematically, such as harmonic progressions, degree of (a)tonality, metrical structure, information content and entropy, music-semantic content, and memory processes. Thus, while the classifier was able to distinguish which class of stimuli was presented, this could have been due to processes other than emotion. However, none of these processes has any known association with the parietal operculum. For example, several studies have investigated neural correlates of processing of musical syntax (such as harmonic progressions or changes in key), and while these studies consistently showed activation of the FOP, none of these studies reported signal changes in primary or secondary somatosensory cortex. The same holds for metrical structure, semantic processes, attention, or memory processes. Thus, these cognitive processes could not sufficiently explain the present results. Future studies could investigate feelings for which music stimuli can be matched more closely (such as heroic- and sad-sounding music)^67^.

## Conclusions

Our decoding results indicate that several neocortical regions significantly encode the neural correlate of subjective feeling. The multivoxel patterns corresponding to feeling representations emerged within seconds, suggesting that future studies can use shorter musical stimuli, and thus also investigate several emotions in one experimental session. Our findings are reminiscent of previous decoding studies, and highlight the importance of the neocortex for the encoding of subjective feelings. In particular, our results indicate that the secondary somatosensory cortex (SII) is a neural substrate of feeling states. We propose that secondary somatosensory cortex (SII, which covers the parietal operculum and part of the posterior insula) synthesizes *emotion percepts* (i.e., preverbal subjective feelings), based on anatomical connections with limbic/paralimbic core structures, sensory-interoceptive and visceromotor structures (insula, ACC), and motor structures (striatum, ventromedial PFC, pre-SMA, PMC). Emotion percepts may be modulated by conscious appraisal or emotion regulation, which involve executive functions, memory, and possibly language. Thus, numerous isocortical regions are involved in the generation and modulation of feeling states. Future studies might take greater care in differentiating insular cortex and SII, and be aware that part of SII is located in the posterior insula.

## Supplementary Information

Supplementary Figure S1.

Supplementary Figure S2.

Supplementary Figure S3.

Supplementary Figure S4.

Supplementary Figure S5.

Supplementary Table S1.

Supplementary Table S2.
