# Supplementary material for: Neocortical substrates of feelings evoked with music in the ACC, insula, and somatosensory cortex
Source: Sci Rep. 2021 May 12;11:10119. doi: 10.1038/s41598-021-89405-y (PMC8115666; doi:10.1038/s41598-021-89405-y)
Supplement: Supplementary file 1 — Supplementary Figure S1. [file 41598_2021_89405_MOESM1_ESM.pdf]

Neocortical substrates of feelings evoked with music in the ACC, insula, and somatosensory cortex

Stefan Koelsch, Vincent K.M. Cheung, Sebastian Jentschke, John-Dylan Haynes

Supplementary Figure S1

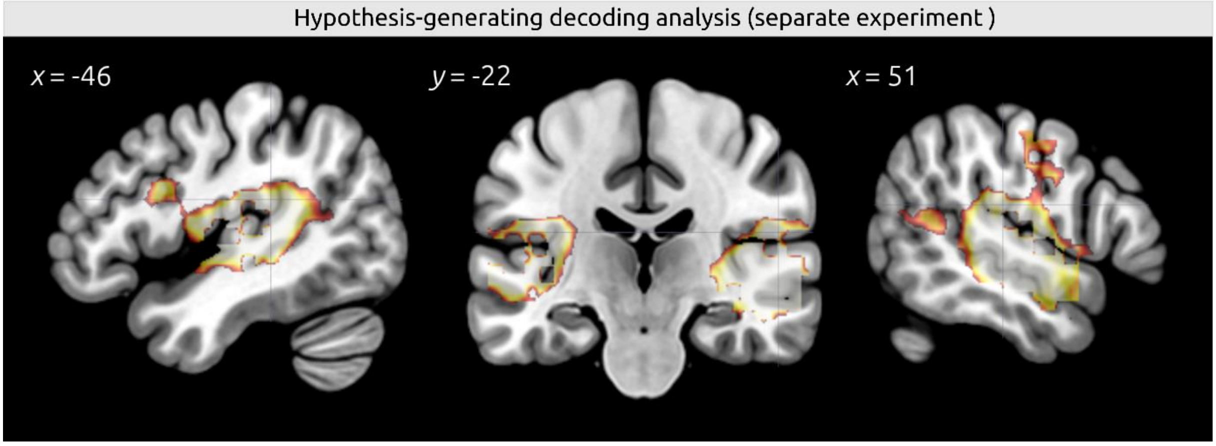

The decoding analysis was performed on data from our previous study [Koelsch, S., Skouras, S., Fritz, T., Herrera, P., Bonhage, C., Küssner, M. B., & Jacobs, A. M. (2013). The roles of superficial amygdala and auditory cortex in music-evoked fear and joy. Neuroimage, 81, 49-60]. Adapting the original design, a general linear model was estimated using SPM12 for each subject with four boxcar regressors (indicating every six presented stimuli) for each music category (joy/neutral/fear), boxcar regressors indicating valence and arousal ratings, as well as boxcar acoustic covariates of no interest, and six motion regressors. All boxcar regressors were convolved with the canonical hemodynamic response function, a 128s high-pass filter was selected, and an AR1 model was used to account for temporal autocorrelations. Binary searchlight decoding was performed on the whole brain between joy and fearful music using support-vector classification implemented with default settings in The Decoding Toolbox v3.99 [Hebart, M. N., Görgen, K. & Haynes, J.-D. (2015). The decoding toolbox (TDT): a versatile software package for multivariate analyses of functional imaging data. Frontiers in neuroinformatics 8, 88]. Classification accuracies were averaged from four-fold cross-validation to control for overfitting. However, please note that independence of each fold is not assumed as the original study was not designed with multivariate decoding in mind. Since the scanner was not stopped throughout the experiment, information from e.g. the first six joyful music presentations may have been carried over to the next six presentations. Results from this preliminary decoding analysis yielded significant clusters with local maxima in the superior temporal gyrus and superior temporal sulcus, posterior insula, parietal operculum, pre- and postcentral gyrus, left frontal operculum (superior pars opercularis) and right posterior middle temporal gyrus. No significant subcortical structures were detected.
