# Supplementary material for: Neocortical substrates of feelings evoked with music in the ACC, insula, and somatosensory cortex
Source: Sci Rep. 2021 May 12;11:10119. doi: 10.1038/s41598-021-89405-y (PMC8115666; doi:10.1038/s41598-021-89405-y)
Supplement: Supplementary file 2 — Supplementary Figure S2. [file 41598_2021_89405_MOESM2_ESM.pdf]

Neocortical substrates of feelings evoked with music in the ACC, insula, and somatosensory cortex

Stefan Koelsch, Vincent K.M. Cheung, Sebastian Jentschke, John-Dylan Haynes

Supplementary Figure S2

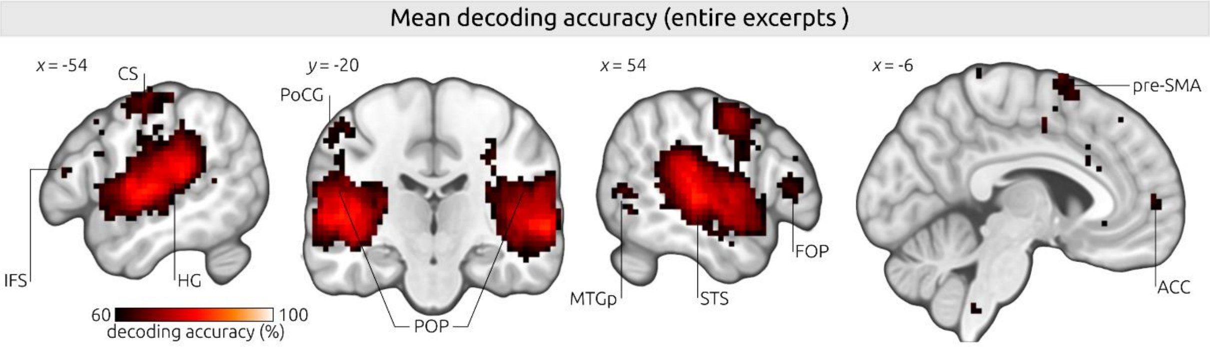

The figure shows the statistical parametric map for the five-fold cross-validated decoding accuracy between joy and fear stimuli, mean-averaged across subjects. As in the main decoding analysis (see main text), no significant decoding results were obtained from subcortical regions such as amygdala, or striatum.
