# Supplementary material for: Neocortical substrates of feelings evoked with music in the ACC, insula, and somatosensory cortex
Source: Sci Rep. 2021 May 12;11:10119. doi: 10.1038/s41598-021-89405-y (PMC8115666; doi:10.1038/s41598-021-89405-y)
Supplement: Supplementary file 3 — Supplementary Figure S3. [file 41598_2021_89405_MOESM3_ESM.pdf]

Neocortical substrates of feelings evoked with music in the ACC, insula, and somatosensory cortex

Stefan Koelsch, Vincent K.M. Cheung, Sebastian Jentschke, John-Dylan Haynes

Supplementary Figure S3

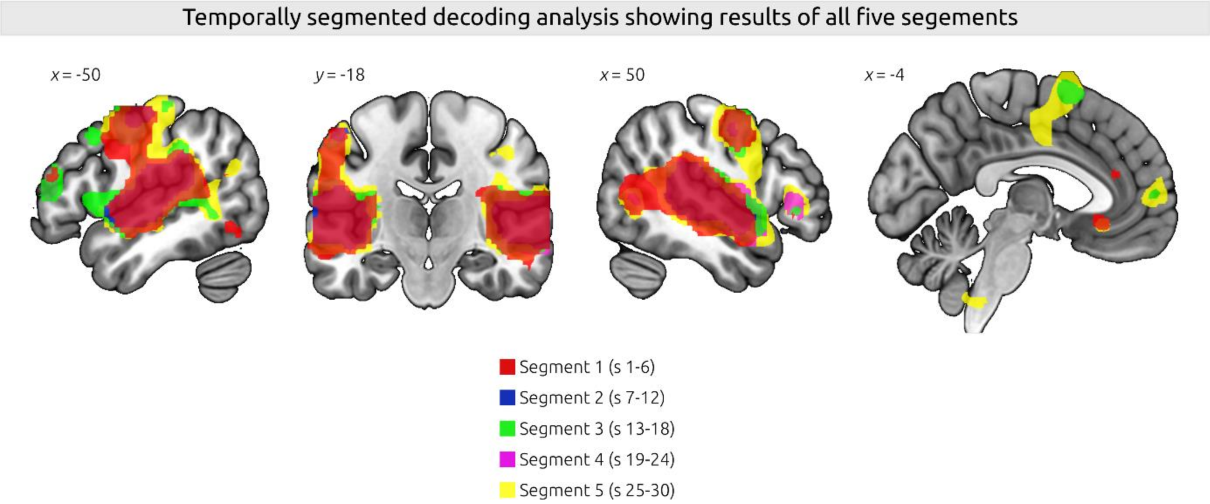

Results of the temporally segmented decoding analysis, separately for all five time segments, showing clusters with voxels providing significant information about the difference between joy- and fear-music (after permutation-based t-tests and correction for multiple comparisons, all results were obtained without spatial smoothing). Note the increase in cluster size across segments in all clusters. For methods see main text.
