# Supplementary material for: Neocortical substrates of feelings evoked with music in the ACC, insula, and somatosensory cortex
Source: Sci Rep. 2021 May 12;11:10119. doi: 10.1038/s41598-021-89405-y (PMC8115666; doi:10.1038/s41598-021-89405-y)
Supplement: Supplementary file 4 — Supplementary Figure S4. [file 41598_2021_89405_MOESM4_ESM.pdf]

Neocortical substrates of feelings evoked with music in the ACC, insula, and somatosensory cortex

Stefan Koelsch, Vincent K.M. Cheung, Sebastian Jentschke, John-Dylan Haynes

Supplementary Figure S4

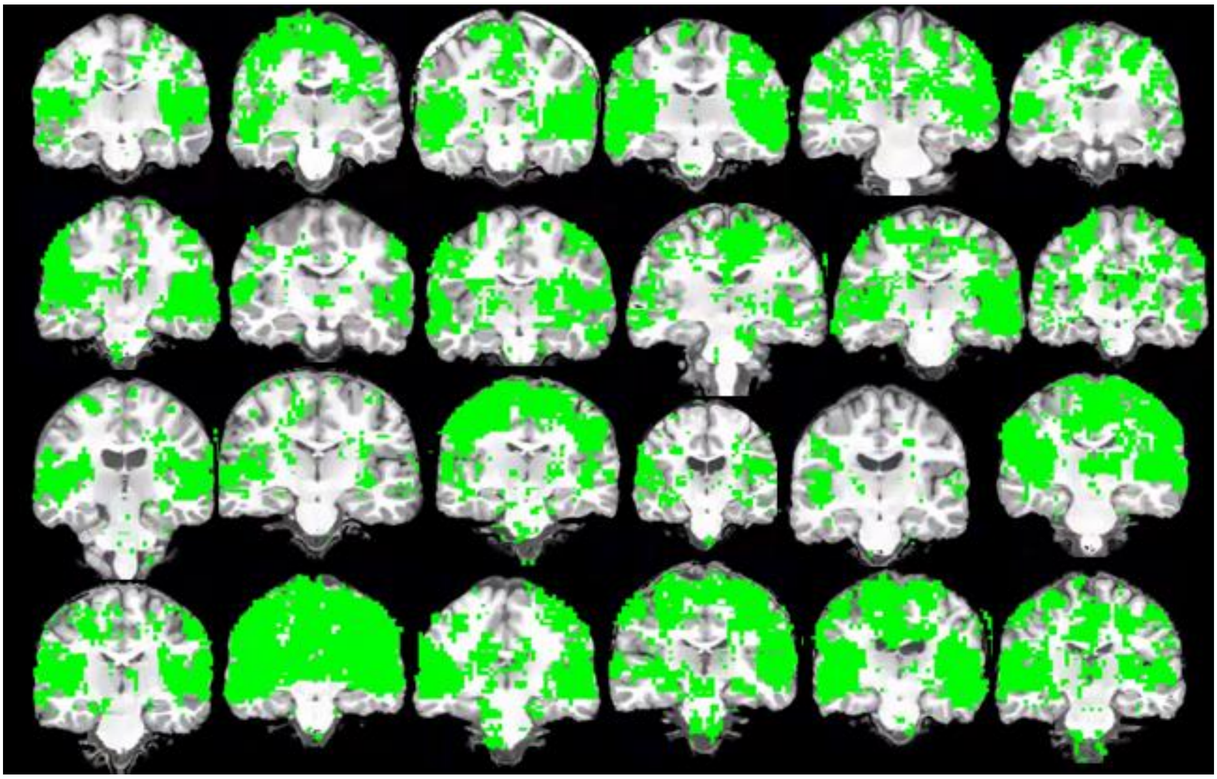

Coronal slices from all 24 participants before normalization into MNI space. Regions in green indicate decoding accuracy of at least 70% in discriminating between joy and fear music stimuli. Note that these regions included the POP bilaterally in 20 participants, and the left POP in 4 participants.
