# Supplementary material for: Neocortical substrates of feelings evoked with music in the ACC, insula, and somatosensory cortex
Source: Sci Rep. 2021 May 12;11:10119. doi: 10.1038/s41598-021-89405-y (PMC8115666; doi:10.1038/s41598-021-89405-y)
Supplement: Supplementary file 5 — Supplementary Figure S5. [file 41598_2021_89405_MOESM5_ESM.pdf]

# Neocortical substrates of feelings evoked with music in the ACC, insula, and somatosensory cortex

Stefan Koelsch, Vincent K.M. Cheung, Sebastian Jentschke, John-Dylan Haynes

**Supplementary Figure S5**

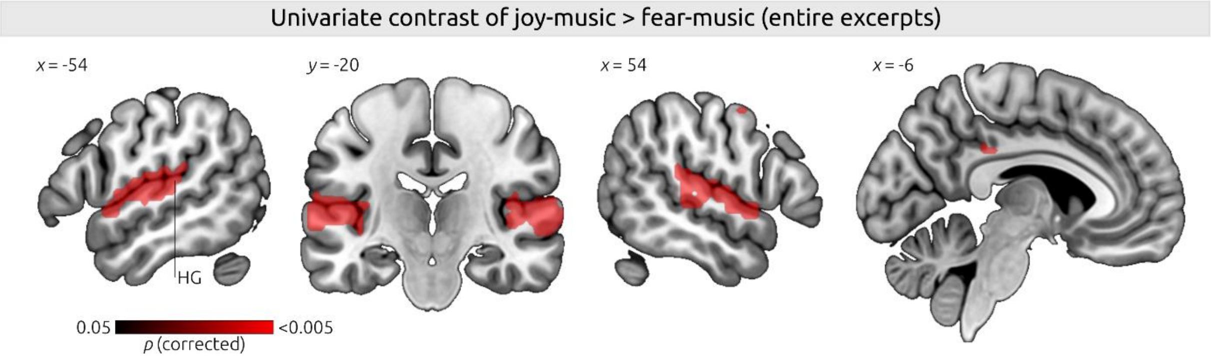


*Statistical parametric map of brain regions showing increased activity for joyful compared to fearful music, analyzed using a mass-univariate approach. Because images were not smoothed during preprocessing, voxel-wise corrections for multiple comparisons were carried out using LISA [Lohmann, G. et al., 2018. LISA improves statistical analysis for fMRI. Nature communications 9, 1–9]. The reverse contrast (fear-music > joy-music) yielded no significant voxels.*
