# Supplementary material for: Neocortical substrates of feelings evoked with music in the ACC, insula, and somatosensory cortex
Source: Sci Rep. 2021 May 12;11:10119. doi: 10.1038/s41598-021-89405-y (PMC8115666; doi:10.1038/s41598-021-89405-y)
Supplement: Supplementary file 6 — Supplementary Table S1. [file 41598_2021_89405_MOESM6_ESM.pdf]

Neocortical substrates of feelings evoked with music in the ACC, insula, and somatosensory cortex

Stefan Koelsch, Vincent K.M. Cheung, Sebastian Jentschke, John-Dylan Haynes

Supplementary Table S1: Overview of sound stimuli

Composer / artist	***Title***	***ASIN Nr.***
Music stimuli evoking fear		
Danny Elfman	The Killing	B00000JC9R
Seiko Kobuchi	Boss Battle (from: Biohazard Zero)	n/a
Michael Giacchino	Monsters Are Such Interesting People (from: Lost)	B000EHSVDM
Michael Giacchino	Charlie’s Dream (from: Lost)	B000I2IQ9M
Ichiro Kohmoto	Unexpected Surprise (from: Resident Evil 0)	n/a
Masami Ueda & Saori Maeda	Cold Sweat (from: Biohazard 3)	B000058A7Y
Music stimuli evoking happiness		
Jaco Pastorius	Soul intro / “The Chicken”	B0000C24JN
Louis Armstrong	St. Louis Blues	B006CBVRJY
Craobh Rua	The Luck Penny	B000003NHN
Alfredo de Angelis	Pregonera	B001P5LDTQ
Orchestra Paraschiv Oprea	Batuta de la Adancata	B00000DTI6
Niccolò Paganini	Violin Concerto No. 1, 3rd movement	B000001GHC
