# Supplementary material for: Neocortical substrates of feelings evoked with music in the ACC, insula, and somatosensory cortex
Source: Sci Rep. 2021 May 12;11:10119. doi: 10.1038/s41598-021-89405-y (PMC8115666; doi:10.1038/s41598-021-89405-y)
Supplement: Supplementary file 7 — Supplementary Table S2. [file 41598_2021_89405_MOESM7_ESM.pdf]

Neocortical substrates of feelings evoked with music in the ACC, insula, and somatosensory cortex

Stefan Koelsch, Vincent K.M. Cheung, Sebastian Jentschke, John-Dylan Haynes

Supplementary Table S2: Statistics of the emotion ratings. The columns “Fear-music” and “Joy-music” provide means (with standard errors of mean in parentheses). Range for the valence, arousal, joy, and fear scales was from -3 (“not at all”) to 3 (“very much”).

	Fear-music	Joy-music	Significance of the difference	
Valence	-1.74 (0.17)	1.59 (0.13)	$F_{(1,24)} = 209.13$; $p < .0001$	***
Arousal	0.24 (0.25)	0.95 (0.21)	$F_{(1,24)} = 3.58$; $p = 0.071$	n.s.
Joy	-1.89 (0.16)	1.62 (0.13)	$F_{(1,24)} = 237.84$; $p < .0001$	***
Fear	0.82 (0.22)	-2.67 (0.10)	$F_{(1,24)} = 187.40$; $p < .0001$	***
